# Statins Decrease Oxidative Stress and ICD Therapies

**DOI:** 10.4061/2010/253803

**Published:** 2010-03-25

**Authors:** Heather L. Bloom, Irfan Shukrullah, Emir Veledar, Rebecca Gutmann, Barry London, Samuel C. Dudley

**Affiliations:** ^1^Division of Cardiology, Department of Medicine, Emory University School of Medicine, Atlanta, GA 30322, USA; ^2^Division of Cardiology, Atlanta Veterans Administration Medical Center, Atlanta, GA 30033, USA; ^3^Section of Cardiology, University of Illinois at Chicago, Chicago, IL 60612, USA; ^4^University of Pittsburg Medical Center Presbyterian, Pittsburg, PA 15213, USA

## Abstract

Recent studies demonstrate that statins decrease ventricular arrhythmias in internal cardioverter defibrillator (ICD) patients. The mechanism is unknown, but evidence links increased inflammatory and oxidative states with increased arrhythmias. We hypothesized that statin use decreases oxidation. *Methods.* 304 subjects with ICDs were surveyed for ventricular arrhythmia. Blood was analyzed for derivatives of reactive oxygen species (DROMs) and interleukin-6 (IL-6). *Results.* Subjects included 252 (83%) men, 58% on statins, 20% had ventricular arrhythmias. Average age was 63 years and ejection fraction (EF) 20%. ICD implant duration was 29 ± 27 months. Use of statins correlated with lower ICD events (*r* = 0.12, *P* = .02). Subjects on statins had lower hsCRP (5.2 versus 6.3; *P* = .05) and DROM levels (373 versus 397; *P* = .03). Other factors, including IL-6 and EF did not differ between statin and nonstatin use, nor did beta-blocker or antiarrhythmic use. Multivariate cross-correlation analysis demonstrated that DROMs, statins, IL-6 and EF were strongly associated with ICD events. Multivariate regression shows DROMs to be the dominant predictor. *Conclusion.* ICD event rate correlates with DROMs, a measure of lipid peroxides. Use of statins is associated with reduced DROMs and fewer ICD events, suggesting that statins exert their effect through reducing oxidation.

## 1. Background

Sudden Cardiac death (SCD) accounts for over 400 000 deaths per year [1] in the United States, more than 50% of all cardiac related death. Ventricular arrhythmias cause most of these deaths [2].The only treatment for ventricular arrhythmias with proven mortality benefit is the internal cardioverter-defibrillator (ICD). Two recent observational trials have demonstrated that Hydroxymethylglutarylcoenzyme A reductase inhibitors (statins) decrease the incidence of ventricular arrhythmias as well as increase survival in patients with ICDs [3, 4]. This survival benefit exists for both ischemic (MADITII) and non-ischemic cardiomyopathies (DEFINITE) and the reduction in ICD discharges is independent of statins' cholesterol-lowering effects.

One proposed mechanism for the antiarrhythmic effect of statins is their antioxidant properties [3]. Statins reduce the generation of reactive oxygen species by inhibition of vascular NAD(P)H oxidase [5, 6], inhibiting the respiratory burst of phagocytes [7], antagonizing the prooxidant effect of angiotensin II and endothelin-1 [8], and increasing the synthesis of vascular nitric oxide [9, 10]. In addition, some statins and their metabolites are direct free radical scavengers. As inflammation is closely linked to the production of reactive oxygen species (ROS), statins may also have important antiinflammatory effects. The molecular basis of these observed antiinflammatory effects of statins may relate to their ability block the production and/or activity of ROS [11].

Several lines of evidence link oxidative stress with arrhythmias [12–14]. H_2_O_2_, a form of oxidative stress, increases ventricular arrhythmias through alterations in cellular electrophysiology reducing sodium channel current and preventing its complete inactivation. The resultant persistent current during the action potential plateau appears to be the result of lipid peroxidation [15]. Patch clamp experiments in rat myocytes have also observed a H_2_O_2_-induced augmentation of sodium current via a slowing of the inactivation kinetics, producing a marked prolongation of the cellular action potential [16]. This provides strong evidence that statins act to reduce arrhythmic risk by reducing lipid peroxidation. Thus we hypothesized that statin medications decrease ventricular arrhythmias by reducing oxidative stress. To test this hypothesis, we compared serum measures of oxidation in the lipid and soluble phases in patients at high arrhythmic risk. This population includes both patients that were taking statins and those that were not, providing a direct measure of the antioxidant effects due to statin use.

## 2. Methods

To select patients at high risk for ventricular arrhythmia, we retrospectively examined patients undergoing either ICD implantation or ICD generator exchange. This study protocol was approved by the Emory University Internal Review Board. These patients were enrolled in the Genetic Risk Assessment for Defibrillator Events (GRADE) trial, and were undergoing new ICD implantation or who had undergone ICD placement or generator exchange within the last 5 years in one of the four Emory University Hospitals. Patients met the inclusion criteria of being age 18 or older, able to give informed consent and having a depressed left ventricular ejection fraction (LVEF) of <30%. Exclusion criteria included patient refusal, patients with a life expectancy less than 6 months, patients who had ongoing class IV heart failure symptoms, patients who were post-cardiac transplant or with left ventricular assist devices. Demographic and medical information obtained on enrollment included: age, gender, race, history of smoking, medications, New York Heart Association (NYHA) class, etiology of heart disease, hypercholesterolemia, history of myocardial infarction (MI) history of coronary artery bypass (CABG) surgery, family history of heart disease, history of arrhythmias, history of syncope, echocardiogram results, cardiac catheterization results, nuclear imaging results, electrocardiograms, blood pressure, heart rate, electrolytes and date of ICD implantation surgery and any ICD generator exchanges. 

### 2.1. Biomarker Data

 A single blood draw was performed at the time of enrollment and analyzed for markers of oxidative stress and inflammation in the Emory Biomarkers Core Laboratory. Markers used to measure oxidative stress were: ratios of oxidized to reduced glutathione (*E*_*h*_ GSH) and cystiene (*E*_*h*_ CySH) in plasma (thiol ratios) [17] and derivatives of reactive oxygen species (DROMs) [18, 19]. Detailed methods to prevent rapid oxidation of samples have been delineated previously [20]. Blood was collected from an antecubital vein and transferred immediately to a micro-centrifuge tube containing 0.5 mL of a preservation solution of 100 mM serine*·*borate (pH 8.5) containing (per mL) 0.5 mg sodium heparin, 1 mg bathophenanthroline disulfonate sodium salt, and 2 mg iodoacetic acid. Use of this procedure minimizes auto-oxidation and hemolysis [20]. All blood was drawn between 7:30 am and 3:00 pm in non-fasting patients. Following centrifugation to remove blood cells, aliquots (200 *μ*L) were transferred to tubes containing 200 *μ*L of 10% (w/v) perchloric acid containing 0.2 M of boric acid and 10 *μ*M *γ*-Glu-Glu as internal standard. Samples were stored at −80°C (<2 months) prior to further processing to form N-dansyl derivatives and analysis by HPLC with fluorescence detection. Reduced glutathione, cystine, and cysteine levels in plasma were greater than 1,000 times the level of detection (*∼*1 nM). Oxidized glutathione levels were approximately 10 times this limit. Previous data have shown stable measurements over this length of storage [20]. Metabolites were identified by coelution with standards, and quantified by integration relative to the internal standard.

We calculate the redox states (*E*_*h*_) of the thiol/disulfide pools using the Nernst equation, *E*_*h*_ = *E*_*o*_ + *R* *T*/*n* *F* ln [disulfide]/[thiol]^2^. *E*_*o*_ is the standard potential for the redox couple, *R* is the Rydberg constant, *T* is the absolute temperature, *n* is 2 for the number of electrons transferred, and *F* is Faraday's constant. The standard potential *E *_o_ used for the glutathione and cystiene redox couples was −264 mV and −250 mV, respectively [20]. Less negative *E*_*h*_ numbers imply a more oxidized state. DROMs were measured in Carr units with higher values indicating higher levels of oxidative stress. DROMs (Diacron International, Grosseto, Italy) and inflammatory markers, high sensitivity C-reactive protein (hsCRP; Life Diagnostics, West Chester, PA), interleukin-1-*β* (IL-1*β*; R&D Systems, Minneapolis, MN), interleukin-6 (IL-6; R&D Systems), and tumor necrosis factor *α* (TNF*α*; R&D Systems), were measured using commercially available kits. The intraassay coefficients of variation (%CV) were *E*_*h*_ GSH, <1 at −156 mV and <1 at −120 mV; *E*_*h*_ CySH, 5.0 at −100 mV and 4.5 at −60 mV; DROMs, 2.2 at 300 Carr units and 2.3 at 550 Carr units; IL-1*β*, 10.1 at 0.2 pg/mL and 5.2 at 10 pg/mL; hsCRP 5.1 at 1 *μ*g/ml and 3.6 at 8 *μ*g/mL; IL-6, 20.9 at 3.2 pg/mL and 6.2 at 50 pg/mL; and TNF*α*, 11.9 at 2 pg/mL and 7.3 at 50 pg/mL.

### 2.2. Ventricular Arrhythmias

 Events were identified through routine device interrogations and chart review. All history of appropriate therapies for ventricular fibrillation (VF) or ventricular tachycardia (VT) were recorded. Dates, times, types and number of therapies were all documented. There was no standardization of ICD programming due to the study design; some patients had antitachycardia pacing (ATP) programmed on while others did not. Thus both ATP and shock therapies were recorded (henceforth referred to as “ICD events”). Independent cardiologists adjudicated therapies as either appropriate therapy for ventricular arrhythmias or inappropriate therapy, for a non-VT/VF. However, as this was a retrospective study, the original strips were not always available in the medical chart and we were unable to re-adjudicate ventricular arrhythmias and had to rely on the original adjudications. Only appropriate therapies documented to be for ventricular arrhythmias were included in the analysis. 

### 2.3. Data Analysis

 Statistical analysis was performed using SPSS software version 14.0 (SPSS Inc., Chicago, Illinois 60606). Baseline characteristics of patients who received and did not receive ICD therapies were compared using a paired *t*-test for continuous variables (expressed as mean ± SD) and Fisher's exact test for categorical variables. Marker data were presented as the mean ± SD, except as noted. In comparing biomarker data based on presence or absence of statin use, we employed the standard t-test for equality of means. All statistical tests were two-tailed with significance taken to be *P* ≤ .05. Patient characteristics and all oxidative and inflammatory markers were examined for links to ICD events using Pearson's correlation coefficients. Multivariate models were used to examine the association between each oxidative marker and the occurrence of ICD therapies while controlling for other inflammatory markers and significant characteristics. Due to the wide range of follow up times, events were examined as a function of time, in “events/months.”

## 3. Results

**Figure 1:**
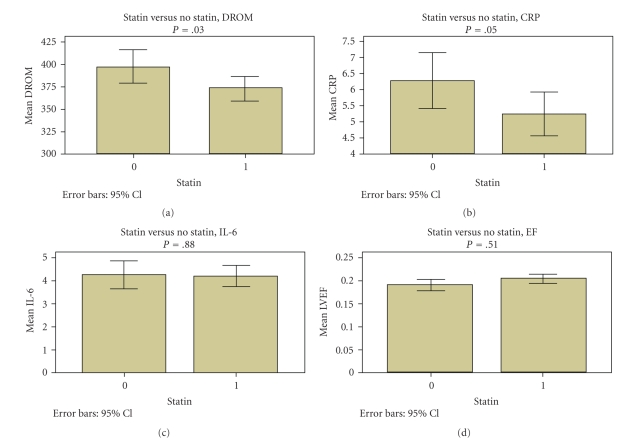
Comparison of mean DROM, CRP, IL-6 and EF by statin use or non-use with respective *P* values. Statin use = 1, non-use = 0.

**Figure 2:**
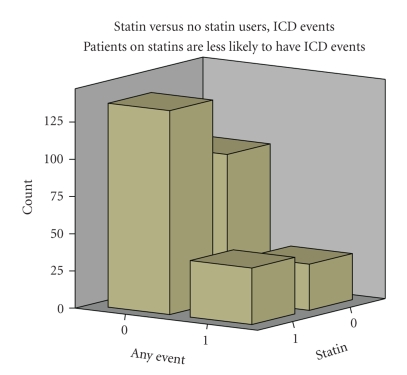
Comparison of events (any events = 1 versus no events = 0) by statin use (= 1) or non-use (= 0). Patients on statins are less likely to experience ICD events.

304 patients were enrolled and had blood tests performed and received at least 3 months of follow up (range: 3 to 135 months, mean 29 months). Table 1 presents their demographic data. There were 252 men (83%) and 52 women (17%). Average age was 63 ± 11, EF 20% ± 7%, 114 (38%) had diabetes, 175 (58%) were on statins, 246 (80%) were on beta-blockers, 61 (20%) were on antiarrhythmics, 236 (77%) had no ICD therapies, 200 (67%) were smokers and 96 (65%) had coronary artery disease (CAD). Other medication use examined included ACE-inhibitors (177, 58%), ARBs (71, 23%) and PPARs (28, 9%), all of which are known to affect oxidative stress. Mean biomarker values were high for all patients (Table 1). Table 2 compares patients using statins to those who were not using statins. Statin users showed significant differences in incidence of CAD (*P* < .01) and cigarette smoking (*P* = .01). Note however, that cigarette smoking correlates directly with CAD and is not an independent variable. Statin users also had significant reductions in DROM and hsCRP. Figure 1 shows EF, hsCRP, DROM and IL-6 by statin use. Table 3 provides the Pearson correlation coefficients and *P* values for the characteristics that correlated with ICD therapy events. Ejection fraction, IL-6 levels, statins, and DROMs each were significant. Figure 2 shows the relationship between statin use and ICD events.

Multivariate cross-correlation analysis confirms the significant relationships of IL-6, DROMs, statins and EF with events. For IL-6: *P* = .024, Pearson coefficient of 0.124; DROM: *P* = .001, Pearson coefficient of 0.183; statins: *P* = .047, Pearson coefficient of −.107; TNF-*α*: *P* = .040, Pearson coefficient of −0.112; and EF: *P* = .015, Pearson coefficient of −0.132. Multivariate linear regression shows DROMs to be the dominant predictive factor of events, with a regression coefficient of 0.164 (*P* = .026). Beta-blockers and antiarrhythmic medications were also examined in the multivariate analysis and were not found to be significant, beta-blockers had a Pearson coefficient of −0.037 (*P* = .54) and antiarrhythmics had a Pearson coefficient of 0.062 (*P* = .29).

## 4. Conclusions

For these high risk patients, we demonstrate that DROM, IL-6 and statins are correlated with decreased ICD event rates. Interestingly, statin use decreased DROM but not IL-6, suggesting IL-6 affects event rates via a separate mechanism. However, DROMs were the dominant predictor of event rate, leaving statin use the primary difference in rate reduction. To further validate our data set, we confirm the previous observation that statin medication use correlates with decreased rates of ventricular arrhythmias as measured by ICD therapies and the independence of ejection fraction as a risk factor for ventricular arrhythmias. When we examined biomarkers to assess inflammation and oxidative stress burden, we found that hsCRP and DROM were decreased in the statin users group and that IL-6 and DROMS correlate with event risk. IL-6 correlates with events, but not with statin use, suggesting that IL-6 is unaffected by statin use. The only factor dependent on statin use and associated with decreased ICD events is DROM. That DROMs are the single most predictive indicator of future events, coupled with their statin correlation, provides strong evidence that the mechanism by which statins lower rates of ventricular arrhythmias is via their antioxidant effect.

## 5. Discussion

Each patient considered had cardiac disease that qualified them for an ICD, giving them a high risk for ventricular arrhythmias. Our patient demographics do not differ significantly from those in the two large trials, previously cited, which demonstrate decreased ICD events with statins. Average patient age, gender, EF, rates of diabetes, and rates of ACE/ARB use were all similar. Perhaps unsurprisingly, a difference was seen in the rates of cigarette smokers. In the ischemic cardiomyopathy group (MADITII), the rate of smoking was 81%, in the non-ischemic group (DEFINITE), 38% were smokers. In our mixed ischemic and non-ischemic population, 67% of patients were smokers. Of our smokers, 76% had CAD (and 73% of our CAD patients were smokers). 

Our patients' biomarkers are elevated. Elevated inflammatory markers and markers of oxidative stress have been correlated with increased mortality in cardiac disease. hsCRP, for example, is considered a “high risk” marker (per AHA/CDC consensus document) [21] when the levels are >3.0 mg/dl. Our mean value was 5.7. In a study recently accepted for publication, we compare case-matched biomarkers for patients with and without atrial fibrillation. In that study we demonstrate that patients with AF are more oxidized compared to the controls. These ICD patients are similarly oxidized when compared our AF patients: DROMS are similar at 388 versus 383 Carr units, *E*_*h*_ C −66 versus −68 mV, *E*_*h*_ G −126 versus −133 mV. For our inflammatory markers hsCRP is higher (5.7 versus 3.9 uGu/ml), as is IL-6 (5.5 versus 4.3 pg/ml), TNF*α* is lower 4.4 versus 6.4 pg/ml, and IL-1*β*, was the same 0.5 versus 0.5 pg/ml. “Normal” values are age and disease state-dependent, but for perspective, the case-matched controls with no arrhythmias in our atrial fibrillation study had mean DROMS 310 Carr units, *E*_*h*_ CySH −77 mV, *E*_*h*_ GSH −154 mV, IL-1*β* 0.4 pg/mL, IL-6 3.9 pg/mL, TNF*α* 5.5 pg/mL, and hsCRP 3.6 *μ*g/mL. 

In these high-risk patients, statin use correlates with decreased arrhythmia risk. Decreases in DROMs correlate with decreased ICD events, and with statin use. This suggests that statin use decreases ventricular arrhythmias in part due to its antioxidant properties, possibly via ion channel alterations.

That IL-6 does not change with statin use has been somewhat controversial in the literature. It has been previously documented to be unchanged with pravastatin, simvastatin, and atorvastatin in several studies [22–24]. Others, however, have seen a change in Il-6 with statin use [25]. As IL-6 is known to exhibit great circadian variation, this particular marker may be more sensitive to the variable followup time courses in our study. However, the lack of correlation with statin use further suggests that statins are acting through an IL-6 independent mechanism. 

Measuring oxidative stress in humans is difficult because free radicals are reactive and thus short-lived. Products of free radical damage to DNA proteins and lipids may provide such markers. Additionally, measurements of O2-generating enzymes can be easily quantified. We chose several markers to examine, quantifying thoi-disulfide redox couples, reduced and oxidized glutathione disulfide, and cystiene/cystine ratios. These redox states represent plasma oxidation state. To reflect the lipid compartment, we used a measure of plasma lipid peroxides known as the DROMS test. The positive correlation of reduced ICD events with DROMS may reflect changes in the lipid compartment, as opposed to the other markers of oxidative stress, which reflect changes in plasma oxidative stress. This finding demonstrates that the tissue oxidative state and the plasma oxidative state are not necessarily equivalent. That DROMS reflect the tissue state, and are significant is further circumstantial evidence to support a tissue-level mechanistic change. 

The mechanisms whereby oxidative stress may contribute to ventricular arrhythmias are unknown, but there is evidence that oxidants can affect ion channel activity [26]. Recently, we have shown that the cardiac sodium channel (SCN5a) promoter region contains an NF-*κ*B response element that could lead to Na^+^ channel transcriptional regulation by a NF-*κ*B-dependent mechanism [27].

Much further research is needed to answer these questions. A prospective examination of the association between oxidative stress markers and ventricular arrhythmias is necessary. Such a prospective study of change in oxidative stress markers in the presence of statin use correlating to decreased ventricular arrhythmias is also warranted.

### 5.1. Limitations

As with all retrospective studies, several limitations exist. The most significant limitation is that we had no consistent time correlation between of our data points; follow up ranged from 3 months to 11 years. There was no correlation between the time of the blood draw for biomarkers and the arrhythmic events. The blood draw was done at the time of enrollment in the study and a patient undergoing generator exchange could have had ICD events that were long before the blood draw was taken. Twenty three percent of our patients experienced ICD events. However, these events could have occurred anytime within 5 years of the blood draw. This weakens the connection between the biomarkers and ventricular events. That statistically significant associations remain despite this limitation suggests that the association is strong. We measured marker levels during usual clinical hours and without fasting, mimicking the most common clinical scenario. Measuring at other times or conditions may affect the results, but there is no known diurnal variation in DROM levels, or hsCRP levels. There does appear to be diurnal variation of plasma reduced thiols related to meals in animals [28], but the effect of this variation on the ratio of oxidized to reduce thiols is unknown. In preliminary studies, diurnal variations in *E*_*h*_ GSH and *E*_*h*_ CySH were small, and have peaks in that are separated by 6–7 hr.

Non-uniformity in ICD programming is another significant limitation. Some patients were programmed as a “shock box”, meaning they would only receive ICD therapy for fast VT or VF, with heart rates in the range of 200+ beats per minute (BPM). Other patients had VT zones set as low as 160 BPM, and may have received therapies (ATP or shock) for slower VT. The rate of VT, however, is an artificial distinction. More patients may have experienced “ventricular arrhythmias” if all patients had an ATP zone programmed. Contrariwise, fewer patients may have received therapy if all patients had been set up as a shock only ICD. This may have shifted a significant number of patients into or out of the therapy group.

Another inconsistency was the lack of quantification of the dose, type, and duration of statin therapy. Our data points are simply binary, indicating statin use or no statin use. Patients were listed as taking statin medications if they were taking any statin medication at the time of study enrollment. If they had been taking a statin for 5 years, but had stopped it the week before enrollment, these patients would be considered as not on a statin. By contrast, patients starting a statin the week prior to enrollment would have been considered as on a statin. We also treat all statin medication types and doses equivalently. Previous studies have noted a “dose effect” of statins [3], and the both the question of dose-response and time-dependent dosing are not accounted for in our model.

These limitations aside, our result is both interesting and thought-provoking. This result provides both a motivation for and a jumping off point to planning future trials to clarify the relationship between statins and arrhythmic events.

## Figures and Tables

**Table 1:** 

Baseline demographics	
Age	62 ± 12
Gender	252 men (83%)
CAD	196 (65%)
DM	114 (38%)
ICD therapies	68 (23%)
Average EF	20% ± 7%
Statins	175 (58%)
Beta-blockers	246 (81%)
Antiarrhythmics	61 (20%)
Smokers	202 (67%)
Afib	87 (29%)
ACE	177 (58%)
ARB	71 (23%)
PPAR	28 (9.2%)

Biomarker	Value

CRP	5.7 ± 4.67
IL-6	4.3 ± 3.2
IL1*β*	0.52 ± 0.37
TNF-*α*	4.4 ± 2.8
DROM	383 ± 95
*E*_*h*_ G	−126 ± 13
*E*_*h*_ C	−66 ± 9

**Table 2:** 

	Statin Use (175)	No Statin Use (129)	*P*
Age	59 ± 13	65 ± 9	.00
Male	146 (83%)	106 (82%)	.46
DM	101 (57%)	40 (31%)	.055
Smokers	127 (72%)	75 (58%)	.01
CAD	138 (78%)	58 (45%)	.00
EF	20 ± 7%	19 ± 7%	.51
Beta-blocker	138 (79%)	105 (81%)	.25
Antiarrhythmic	30 (17%)	31 (24%)	.11
Afib	53 (30%)	50 (29%)	.87
CRP	5.2 ± 4.4 ug/ml	6.3 ± 5.0 ug/ml	.05
DROM	373 ± 87 Carr	397 ± 102 Carr	.03
IL-*β*	0.52 ± 0.37 pg/ml	0.53 ± 0.36 pg/ml	.90
IL-6	4.3 ± 3.4 pg/ml	4.5 ± 3.0 pg/ml	.88
TNF-*α*	4.5 ± 3.0 pg/ml	4.3 ± 2.6 pg/ml	.64
*E*_*h*_ G	−126 ± 12 mV	−126 ± 13 mV	.82
*E*_*h*_ C	−66 ± 9 mV	−67 ± 9 mV	.87

**Table 3:** 

ICD events*	Pearson's Correlation Coefficient	*P* value
Age	−0.058	.37
Gender	−0.033	.57
DM	0.052	.37
Cigs	0.079	.17
CAD	0.37	.52
EF	−0.120	.04
Beta-Blocker	−0.037	.54
AntiArrhythmic	0.062	.29
CRP	0.057	.37
DROM	0.188	.003
IL-6	0.129	.043
IL-*β*	−0.065	.30
TNF-*α*	−0.111	.08
*E*_*h*_ GSH	0.064	.32
*E*_*h*_ CYS	−0.005	.94
Statin	−0.114	.037

*Analysis by event-months.
